# Valproate MHRA Guidance: Limitations and Opportunities

**DOI:** 10.3389/fneur.2019.00139

**Published:** 2019-02-20

**Authors:** Lance Watkins, Hannah Cock, Heather Angus-Leppan, Kim Morley, Mike Wilcock, Rohit Shankar

**Affiliations:** ^1^Mental Health and Learning Disability Delivery Unit, Llwyneryr Unit, Neath Port Talbot CLDT, Abertawe Bro Morgannwyg University Health Board, Swansea, United Kingdom; ^2^Atkinson Morley Regional Neuroscience Centre, St George's University Hospitals NHS Trust, London, United Kingdom; ^3^Institute of Medical and Biomedical Education, St George's University of London, London, United Kingdom; ^4^Epilepsy Initiative Group, Royal Free London, London, United Kingdom; ^5^University College, London, United Kingdom; ^6^Centre for Research in Public Health and Community Care, University of Hertfordshire, London, United Kingdom; ^7^Department of Obstetrics and Gynaecology, Royal Hampshire County Hospital, Hampshire Hospitals NHS Foundation Trust, Winchester, United Kingdom; ^8^Royal Cornwall Hospitals Trust, Truro, United Kingdom; ^9^Cornwall Partnership NHS Foundation Trust, Threemilestone Industrial Estate, Truro, United Kingdom; ^10^Exeter Medical School, Knowledge Spa, Royal Cornwall Hospital, Truro, United Kingdom

**Keywords:** valproate, pregnancy, communication, risk assessment, intellectual disability

## Abstract

Recent publication of the Medicines and Healthcare products Regulatory Agency (MHRA) in the United Kingdom has strengthened the regulatory measures for valproate medicines. It highlights the importance of making women of childbearing age with epilepsy aware of the teratogenic risks of valproate and encourages the withdrawal of it from those currently prescribed. While a significant directive, it raises concerns of not having considered the impact on special populations such as women with Intellectual Disability (ID). While it is important that women with ID are not excluded from such safety initiatives, due caution needs to be taken on a case by case basis preferably, to ensure their best interests are central to the decision making. Many women with moderate to profound ID cannot have informed consented sexual relationships not to mention cognitive incapability to make informed choices on medication suitability. These women are at potential risk of having their epilepsy control undermined due to the MHRA directives. Around 30% of people with moderate to profound ID have seizures of which 60% are considered treatment resistant. In this vulnerable population changes to medication without clear clinical and social insights could lead to increased harm levels. This paper enumerates the challenges of application of the new directive to these special populations and proposes a pathway based on individual cognitive ability to provide informed consent to facilitate the continuation or removal of valproate. It is important not to lose sight of individual circumstances and the importance of working collaboratively toward providing person center care.

## Introduction

The Medicines and Healthcare products Regulatory Agency (MHRA) in the United Kingdom have recently strengthened the regulatory measures for valproate medicines (1). The new regulations contraindicate the use of valproate medicines in girls or women of child bearing age unless they participate in the Pregnancy Prevention Programme (Appendix 1).

The MHRA regulations recognize that it is not safe for some women to discontinue valproate prescription in pregnancy and doing so may in fact pose considerable risk to both the mother and the fetus. In such scenarios it may be more appropriate to continue treatment under specialist care (1). There are however exceptional circumstances and practical implications that have not been fully considered by the MHRA regulations. Notwithstanding the requirement for clear documentation and informed consent, it is important not to lose sight of individual circumstances and the importance of working collaboratively toward providing person center care (2).

## Valproate-evidence of Teratogenic Risk

Valproate is a known serious teratogen associated with a risk of major congenital malformations (MCM) (Table A1). A MCM is defined as an abnormality of an essential anatomical structure interfering with function or requiring major intervention (3). The evidence base for valproate teratogenicity is largely based on observational studies of people with epilepsy.

In addition to the MCM risk in children of mothers prescribed valproate there is an increased association with neurodevelopmental, cognitive, and behavioral sequelae. Intelligence Quotient (IQ) assessments have consistently shown lower scores associated with valproate exposure in comparison to other antiepileptic drugs (AEDs). The Neurodevelopmental Effects of Antiepileptic Drugs (NEAD) study was a prospective observational study in which cognitive assessments were completed on children at a 6 year interval. The assessors were unaware of which antiepileptic drug was prescribed for the mother in each case. Valproate exposure was associated with lower IQ scores than other commonly prescribed AEDs (Lamotrigine, Carbamazepine, and Phenytoin). This was evidenced across domains of verbal ability, non-verbal ability, memory, and executive function. Adverse effects on cognition were more prominent in association with high dose valproate prescription which was not observed in the other AEDs investigated (4). This cohort also demonstrated statistically significant lower scores on a range of adaptive functioning assessments, and a specific increase in association with attention deficit hyperactivity disorder (ADHD) (5). A population-based study of all live births in Denmark for 1996–2006 compared exposure to valproate to autism spectrum disorder (ASD) diagnosis. The relative risk of ASD diagnosis in this population was over 4% (6).

Both with respect to the MCMs, and the neurodevelopmental concerns, there appear to be a clear dose dependent relationship (5, 7–9). A family history of MCMs or neurodevelopmental problems contributes to the risk on an individual basis. Therefore, women prescribed low dose valproate without additional risk factors may be at lower risk. The totality of the long-term risks associated with valproate remains unknown as regulatory bodies have not followed the trajectory of development and health in all children exposed to this teratogen during pregnancy.

## Valproate Prescription in Exceptional Circumstances

For some women of child-bearing age on valproate treatment participation in the Pregnancy Prevention Programme may not be appropriate (Appendix 2). In these scenarios there are still situations in which a Risk Acknowledgment Form will need to be signed by the individual and specialist following informed discussion. However, the current Risk Acknowledgment Form does not allow for these circumstances. For a woman who is trying to conceive, or one who is sexually abstinent the “Effective contraception is essential while taking valproate,” and specific emphasis on invasive methods (coil, implants or sterilization) is clearly inappropriate and potentially discriminatory. Another vulnerable group of women are those who lack capacity to fully participate in the decision making process. In these scenarios the only option may be for the specialist to amend the form by hand and justify those areas that are not applicable.

## Valproate in People With Epilepsy and Intellectual Disability

People with epilepsy and ID require specific consideration. The prevalence of epilepsy in people with ID is far higher than the general population (10). The etiological influences are complex and include genetic abnormalities (including epilepsy syndromes), underlying structural changes, and co-morbid neurological deficits (11). This complexity contributes to the fact that people with epilepsy and ID are refractory to antiepileptic medication. A population-based prevalence study has shown that up to 68% of people with epilepsy and ID may be treatment resistant (12). Therefore, any change in medication will require careful consideration for those individuals who are stable on monotherapy valproate or a combination of valproate and other AEDs. In addition, people with ID and epilepsy commonly suffer psychiatric co-morbidities (13, 14). Therefore, valproate is often considered a first line treatment for its additional mood stabilizing benefits to help moderate the pharmacological load.

The evidence base for prescribing antiepileptic medication for people with epilepsy and ID is limited and there are no randomized controlled trials or Class I evidence (15). The prescription of valproate in this population is largely guided by evidence extrapolated from large robust investigations in the general population such as the SANAD study (16). Valproate remains a first-line drug for generalized seizures, and there is also evidence that people with ID and treatment resistant seizures may be more responsive (15).

## Consent

Where an individual lacks capacity to make informed decisions around their antiepileptic medication a formal best interest's process should be followed (Pathway A1). If lack of capacity is temporary (emergency situation) the individual should be notified of treatment and if treatment continues, informed consent should be obtained at the earliest opportunity.

A proportion of women with ID and epilepsy will permanently lack capacity to make informed decisions about their medical care including the prescription of AEDs. Capacity should be assessed formally for each decision as set out in the guidance from the Mental Capacity Act (MCA) (2005). In accordance with the principles of the MCA, all practicable steps should be taken to engage the individual in the decision making process. This includes making reasonable adjustments and the use of communication aids and/or assistance from specialist speech and language therapists. Impaired capacity may include difficulty understanding the risk and benefits of the medication; difficulties in retaining relevant information for long enough to make a decision; difficulties in weighing up relevant information; difficulties in communicating any decision.

Where individuals are assessed and deemed to lack capacity to make an informed decision, it is important to attempt to understand their views and involve the individual in decision making process where possible and appropriate. This could be facilitated through purposely designed valproate resources for the ID community, and the opportunity for advocacy support to help the individual make their voice heard and challenge decisions. Family and caregivers are an important source of information, however, it is essential to appreciate the vulnerability of this population. There is an inherent risk of decisions being imposed on individuals without any involvement in the decision making process.

In the MHRA regulations there are two clear decisions that require informed consent-valproate treatment, and participation in the Pregnancy Prevention Programme. Capacity to provide informed consent should be assessed for these two decisions individually. Many women with more moderate to profound ID will not only lack capacity to consent to medical treatment but also lack capacity to consent to sexual relationships. Participation in the Pregnancy Prevention Programme includes adherence to invasive contraceptive methods. These contraceptive methods are associated with their own risks and therefore participation may not be in an individual's best interest. For individuals who lack capacity to consent to sexual relationships pregnancy would raise serious safeguarding concerns, implying sexual abuse. Approaching discussion around this topic unnecessarily can potentially be very distressing for the individual, their families, and caregivers.

## Conclusion

The regulatory measures for valproate prescription from the MHRA are based on a weight of evidence that has guided clinical practice for some time. However, there are individuals and scenarios that have not been fully considered in the regulation. There are women who may consider the contraceptive methods advised by the MHRA unacceptable for personal, religious, or health reasons. In such circumstances the regulations discriminate against these women who are in a position to make fully informed decisions around treatment choice. The MHRA guidance to only prescribe valproate in rare cases for patients who are resistant or intolerant of other treatments does not consider the complex reality of clinical practice including:

1. Women who have not trialed an alternative antiepileptic drug nor do they wish to for fear of potential life threatening consequences of uncontrolled seizures, therefore their response to other treatment is unknown.
2. Women under-going a valproate treatment change in pregnancy-a slow titration process where the consequences of polytherapy and impact on teratogenicity and seizure control are largely unknown.

The regulations neglect to consider women who lack capacity to make informed decisions around medical care. This includes some women with ID who are more likely to have treatment resistant seizures and multiple physical and psychiatric co-morbidities. There are also practical considerations that will impact on primary care services, community pharmacists and secondary services. People on stable doses of valproate, with and without capacity, and even where there is recent and comprehensive documentation about counseling and decision making, are now being referred back into already pressured secondary care services to complete a Risk Acknowledgment Form. All now require annual specialist review purely for this purpose. Once the form is completed (including following a best interest's process for individuals who lack capacity) where prescription of valproate continues, there should be no need to change existing arrangements for prescribing undertaken in primary care. For those who lack capacity, where a best interest decision is established and discussions with the community pharmacist completed- this should be placed on an individual's patient medication record to prevent further unnecessary questioning every time valproate is dispensed which could cause inappropriate distress. In adhering to the MHRA guidelines with the current Risk Acknowledgment Form it is challenging to deliver person center care. Without modification, the current Risk Acknowledgment Form does not recognize scenarios where enforced invasive contraception maybe inappropriate, and in which individuals lack capacity to consent to treatment where a Best Interest process is required. In fact it is in contradiction to the MCA (2005) as no individual has the authority to sign the form to consent on behalf of another adult, unless there are formal legal powers in place.

## Author Contributions

LW: preliminary draft. HC, HA-L, KM, and MW: conceptualization, editing, and redrafting. RS: supervision, process management, editing, proofreading, and submission.

### Conflict of Interest Statement

HC reports in the last 3 years non-financial support from European Academy of Neurology; personal fees from Sage Pharmaceuticals Ltd, Eisai Europe Ltd, UCB Pharma Ltd, European Medicines Agency, from UK Epilepsy Nurse Specialist Association, non-financial support from Special Products Ltd, outside the submitted work. HA-L has been an Association of British Neurologists representative on the MHRA Valproate Stakeholders' Network meeting (2018) and UK representative on the Sanofi European Valproate educational programme Advisory Board (2018). She holds Eisai Investigator initiated non-pharmaceutical grants (2017) and has received Honoraria for non-promotional lectures from Eisai (2017) and UCB (2016). RS is a principal stakeholder of the “SUDEP and Seizure Safety Checklist,” a developer of EpSMon. He has received institutional and research support and personal fees from LivaNova, UCB, Eisai, Veriton Pharma, Bial and Desitin outside the submitted work. The remaining authors declare that the research was conducted in the absence of any commercial or financial relationships that could be construed as a potential conflict of interest.

## References

[1] Medicines and Healthcare Products regulatory Agency New Measures to Avoid Valproate Exposure in Pregnancy. Available online at: https://www.gov.uk/drug-safety-update/valproate-medicines-epilim-depakote-contraindicated-in-women-and-girls-of-childbearing-potential-unless-conditions-of-pregnancy-prevention-programme-are-met (2018).

[2] Angus-Leppan H Shankar R Cock H. Valproate, women, and exceptional circumstances. BMJ (2018) 362:k3625. 10.1136/bmj.k3625 30154117

[3] Pennell PB. Use of antiepileptic drugs during pregnancy: evolving concepts. Neurotherapeutics (2016) 13:811–20. 10.1007/s13311-016-0464-0 27502786 PMC5081129

[4] Meador KJ Baker GA Browning N Cohen MJ Bromley RL Clayton-Smith J. Fetal antiepileptic drug exposure and cognitive outcomes at age 6 years (NEAD study): a prospective observational study. Lancet Neurol. (2013) 12:244–52. 10.1016/S1474-4422(12)70323-X 23352199 PMC3684942

[5] Cohen MJ Meador KJ Browning N May R Baker GA Clayton-Smith J Fetal antiepileptic drug exposure: adaptive and emotional/behavioral functioning at age 6 years. Epilepsy Behav. (2013) 29:308–15. 10.1016/j.yebeh.2013.08.001 24012508 PMC3902100

[6] Christensen J Grønborg TK Sørensen MJ Schendel D Parner ET Pedersen LH. Prenatal valproate exposure and risk of autism spectrum disorders and childhood autism. JAMA (2013) 309:1696–703. 10.1001/jama.2013.2270 23613074 PMC4511955

[7] Bromley R Weston J Adab N Greenhalgh J Sanniti A McKay AJ Treatment for epilepsy in pregnancy: neurodevelopmental outcomes in the child. Cochrane Database Syst Rev. (2014) 10:CD010236 10.1002/14651858.CD010236.pub2 PMC7390020 25354543

[8] Vajda FJ O'brien TJ Graham JE Lander CM Eadie MJ. Dose dependence of fetal malformations associated with valproate. Neurology (2013) 81:10–212. 10.1212/WNL.0b013e3182a43e81. 23911758

[9] Campbell E Kennedy F Russell A Smithson WH Parsons L Morrison PJ. Malformation risks of antiepileptic drug monotherapies in pregnancy: updated results from the UK and Ireland Epilepsy and Pregnancy Registers. J Neurol Neurosurg Psychiatry (2014) 85:1029–34. 10.1136/jnnp-2013-306318 24444855

[10] Robertson J Hatton C Emerson E Baines S. Prevalence of epilepsy among people with intellectual disabilities: a systematic review. Seizure (2015) 29:46–62. 10.1016/j.seizure.2015.03.016 26076844

[11] Busch RM Najm I Hermann BP Eng C. Genetics of cognition in epilepsy. Epilepsy Behav. (2014) 41:297–306. 10.1016/j.yebeh.2014.05.026 24973143 PMC4268334

[12] McGrother CW Bhaumik S Thorp CF Hauck A Branford D Watson JM. Epilepsy in adults with intellectual disabilities: prevalence, associations and service implications. Seizure (2006) 15:376–86. 10.1016/j.seizure.2006.04.002 16782360

[13] Turky A Felce D Jones G Kerr M. A prospective case control study of psychiatric disorders in adults with epilepsy and intellectual disability. Epilepsia (2011) 52:1223–30. 10.1111/j.1528-1167.2011.03044.x 21453357

[14] Beavis J Kerr M Marson AG Pharmacological interventions for epilepsy in people with intellectual disabilities. Cochrane Database Syst Rev. (2005) 9:CD005399 10.1002/14651858 17636795

[15] Doran Z Shankar R Keezer MR Dale C McLean B Kerr MP. Managing anti-epileptic drug treatment in adult patients with intellectual disability: a serious conundrum. Eur J Neurol. (2016) 23:1152–7. 10.1111/ene.13016 27106363

[16] Marson AG Al-Kharusi AM Alwaidh M Appleton R Baker GA Chadwick DW. The SANAD study of effectiveness of valproate, lamotrigine, or topiramate for generalised and unclassifiable epilepsy: an unblinded randomised controlled trial. Lancet (2007) 369:1016–26. 10.1016/S0140-6736(07)60461-9 17382828 PMC2039891

[17] Hernández-Díaz S Smith CR Shen A Mittendorf R Hauser WA Yerby M. Comparative safety of antiepileptic drugs during pregnancy. Neurology (2012) 78:1692–9. 10.1212/WNL.0b013e3182574f39 22551726

[18] Angus-Leppan H Liu RS. Weighing the risks of valproate in women who could become pregnant. BMJ (2018) 361:k1596. 10.1136/bmj.k1596 29669717

[19] Tomson T Battino D Bonizzoni E Craig J Lindhout D Perucca E. Comparative risk of major congenital malformations with eight different antiepileptic drugs: a prospective cohort study of the EURAP registry. Lancet Neurol. (2018) 17:530–8. 10.1016/S1474-4422(18)30107-8 29680205

[20] Vajda FJ O'brien TJ Lander CM Graham J Eadie MJ. The teratogenicity of the newer antiepileptic drugs–an update. Acta Neurologica Scandinavica. (2014) 130:234–8. 10.1111/ane.12280 25040242

[21] Shankar R Bradley M Jory C Olotu V Consent to contraceptive treatment among clients with epilepsy. Learn Disabil Pract. (2013) 16:27–30. 10.7748/ldp2013.12.16.10.27.e1485

[22] Vajda FJ O'Brien TJ Hitchcock A Graham J Cook M Lander C. Critical relationship between sodium valproate dose and human teratogenicity: results of the Australian register of anti-epileptic drugs in pregnancy. J Clini Neurosci. (2004) 11:854–8. 10.1016/j.jocn.2004.05.003 15519862

