# Supplementary material for: Valproate MHRA Guidance: Limitations and Opportunities
Source: Front Neurol. 2019 Feb 20;10:139. doi: 10.3389/fneur.2019.00139 (PMC6391862; doi:10.3389/fneur.2019.00139)
Supplement: Supplementary file 1 [file Data_Sheet_1.pdf]

Appendix I Participation in the Pregnancy Prevention Programme for women of child-bearing age prescribed valproate [1].

1. The individual being informed of and understanding the risks of valproate use in pregnancy, and signing a Risk Acknowledgement Form along with a specialist.
2. Annual review with a specialist.
3. Adherence to a highly effective but invasive contraception, with a pregnancy rate of less than 1%.

Appendix 2 Consensus opinion of exceptional circumstances for valproate prescription in women of childbearing age [2]

1. *Emergency treatment*- A specialist may consider valproate the most effective medication to manage uncontrolled seizure activity or status epilepticus. In emergency situations it may not be possible to obtain informed consent. Best interest decisions are made rapidly by treating clinicians based on evidence and need. On retaining capacity individuals should be informed of the treatment given and if valproate treatment continues informed consent with participation in the Pregnancy Prevention Programme should be attained at the earliest opportunity.
2. *Informed consent*- There will be women of a childbearing age in whom after a fully informed discussion choose to continue with valproate prescription and do not wish to participate in the Pregnancy Prevention Programme [19]. This includes women who are actively looking to conceive with that decision supported by a specialist.
3. *Lack of capacity*- There will be a population of individuals who lack capacity to make informed choices around medication; this may include a proportion of women with intellectual disability (ID). People with moderate to profound ID may also lack capacity to consent to sexual relationships and participation in the Pregnancy Prevention Programme. Adherence to invasive contraceptives may put women in this population at unnecessary risk to physical and emotional wellbeing, and be arguably unethical.

Table I. Major malformation rates associated with valproate in prospective observational studies of National/International pregnancy registries

**International pregnancy register**	**Time period** (valproate sample)	**Major malformation rate**
North American AED pregnancy register [17]	1997-2011 (323)	9.3%
Australian pregnancy registry [8]	1999-2010 (436)	13.8%
UK and Ireland pregnancy register [9]	1996-2012 (1290)	6.7%
International registry of Antiepileptic drugs and Pregnancy (EURAP) [18]	1999-2016 (1381)	10.3%

Pathway I. Flow chart for valproate prescription where individulas lack capacity to provide informed consent [16].

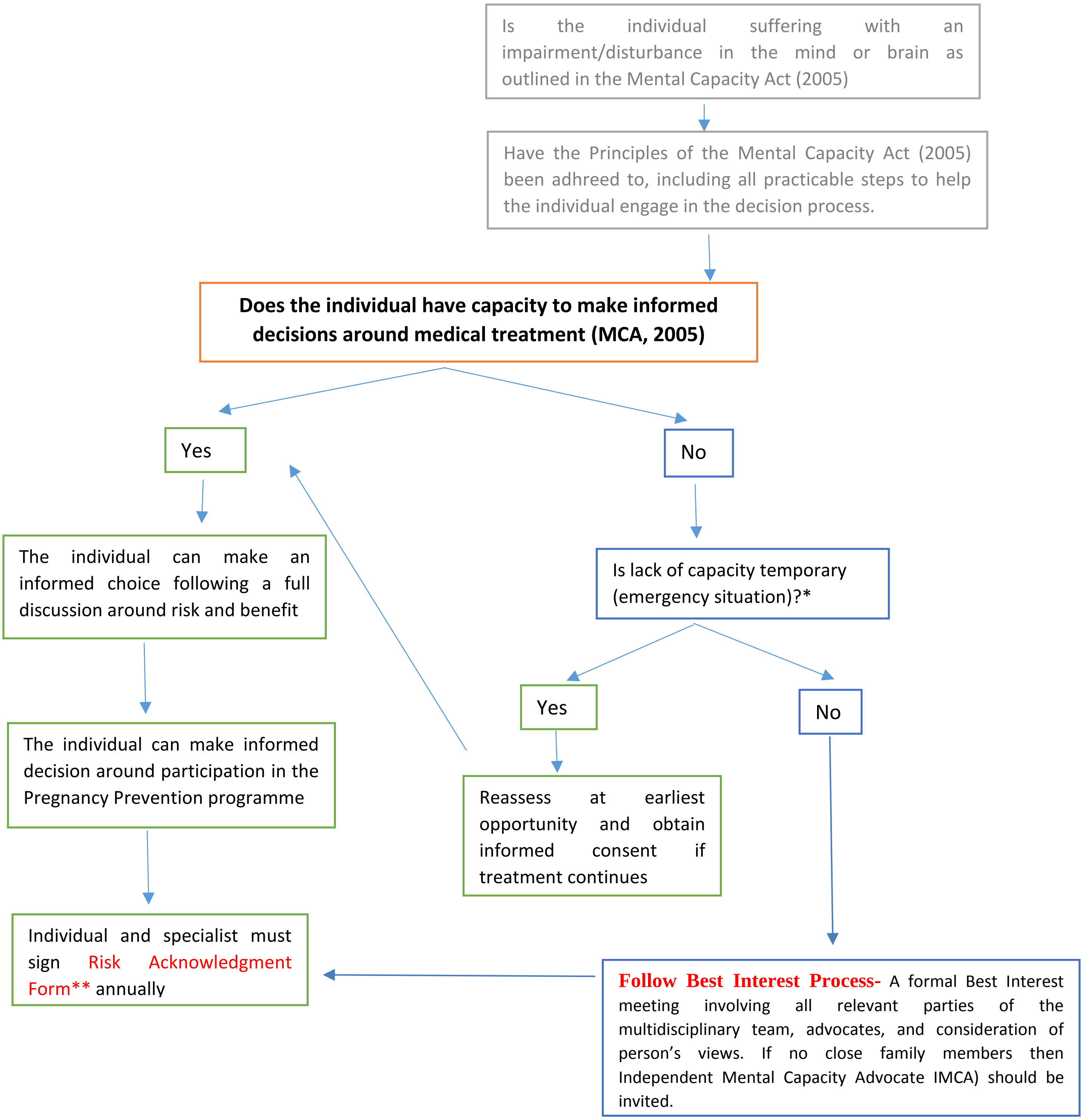

*In an emergency situation a formal best interest process may not be possible see Fig III

** annotated by clinician as required to suit individual circumstances if indicated
